# Alfalfa *MsSOS2* confers salinity tolerance by promoting lateral root growth and regulating Na^+^/K^+^ homeostasis

**DOI:** 10.1038/s41598-025-21355-1

**Published:** 2025-12-04

**Authors:** Biswa R. Acharya, Lorenso A. R. Reyes, Lipsa Mishra, Jorge F. S. Ferreira, Todd H. Skaggs, Devinder Sandhu

**Affiliations:** 1 USDA-ARS, US Salinity Lab, 450 W Big Springs Road, Riverside, CA 92507 USA; 2 College of Natural and Agricultural Sciences, University of California Riverside, 900 University Avenue, Riverside, CA 92521 USA

**Keywords:** Alfalfa, *Medicago sativa*, Salinity, Salt stress, SOS2, SOS pathway, Genetics, Agricultural genetics

## Abstract

Salinity is a major abiotic stress limiting plant productivity. The Salt Overly Sensitive (SOS) pathway, comprising SOS1, SOS2, and SOS3, is central to ion homeostasis under salt stress. While the pathway has been extensively studied in model plants, its components remain uncharacterized in *Medicago sativa* (alfalfa), a moderately salt-tolerant forage crop of global economic importance. Here, we report the functional characterization of *MsSOS2*, a *CIPK* family gene and putative ortholog of *AtSOS2*. Sequence analysis revealed that MsSOS2 possesses conserved domains essential for kinase activity, including a kinase domain, activation loop, the phosphatidic acid (PA)-binding site, and NAF motif. Phylogenetic analysis placed MsSOS2 with AtSOS2 and OsSOS2. Yeast-two-hybrid assays confirmed the interaction between MsSOS2 and MsSOS3, mimicking the AtSOS2*–*AtSOS3 complex. Transgenic expression of *MsSOS2* in the *Arabidopsis sos2* mutant restored salinity tolerance during seed germination, seedling development, and late vegetative growth. Ion profiling showed reduced Na accumulation and enhanced K retention in *MsSOS2*-expressing lines under salinity. We propose that MsSOS2 activates AtSOS1 to facilitate Na⁺ efflux and disrupts AtCBL10-mediated inhibition of AKT1 to promote K⁺ uptake. This study provides the first functional validation of an SOS pathway component in alfalfa and highlights *MsSOS2* as a promising candidate for improving salinity tolerance in legumes.

**Supplementary Information:**

The online version contains supplementary material available at 10.1038/s41598-025-21355-1.

## Introduction

Salinity is one of the most severe abiotic stressors affecting plant growth, development, and yield. Excessive salt in soil or irrigation water disrupts physiological processes, ultimately reducing agricultural productivity and threatening global food security^1^. Increasing salinity in soils and water resources is driven by both natural processes and anthropogenic factors^2^. An overabundance of salt in the soil negatively impacts soil structure by decreasing porosity, which in turn contributes to decreased water uptake by plants. Soil salinity affects more than 800 million hectares of irrigated land and is expected to worsen due to continued expansion of irrigation into marginal lands and environmentally unsustainable water management practices^3^. Salinity adversely affects the growth and productivity of both monocot and dicot species, with the severity of impact varying according to their inherent tolerance levels^4,5^.

Plants have evolved mechanisms to modulate their root development and growth patterns in response to various stresses for optimizing resource uptake and enhancing survivability. Roots are extremely sensitive to their surrounding environment, and their response to salinity stress is both complex and dynamic in nature^6^. Salinity-induced osmotic stress disrupts water balance, impairs metabolic functions, and reduces turgor pressure, thereby inhibiting cell division, cell elongation and overall root expansion^7,8^. Salt stress impedes plant development across multiple stages, from seed germination and seedling growth to the late vegetative stage of plant growth and biomass accumulation^9,10^. Although plants are sessile organisms they possess adaptive responses to environmental stresses, such as salinity. For example, under mild salt stress (e.g., 50 mM NaCl), wild-type Arabidopsis seedlings show a proliferation of lateral roots, a phenomenon known as stress-induced morphogenic response, which is thought to help the plant tolerate salinity^11^.

Based on salinity tolerance, plants are divided into two primary categories: glycophytes, which are sensitive to salt, and halophytes, which are tolerant to salt. Halophytes can thrive in environments with NaCl concentrations exceeding 200 mM, a level at which glycophytes typically fail to survive^12^. Interestingly, some halophytes not only tolerate but also exhibit enhanced growth under low salinity compared to non-saline conditions.

Calcium sensors play important roles in both sensing Ca^2+^ and transducing the signal in various cellular processes^13^. Plants have various calcium sensors, including CBLs (calcineurin B-like). Upon sensing calcium, CBL binds to it and interacts with CIPK partner(s), which in turn allows the CBL-CIPK complex to phosphorylate the target protein, leading to the modulation of the target protein’s biochemical activity. In Arabidopsis, CBL4 (also known as salt overly sensitive 3 or SOS3), CBL8, and CBL10 contribute to salinity tolerance^14^.

CBL-interacting protein kinases (CIPKs) are a family of plant-specific serine/threonine protein kinases that play crucial roles in various aspects of plant life. In *Arabidopsis thaliana*, out of the 26 identified CIPKs, only two—CIPK24 (also known as salt overly sensitive 2 or SOS2) and CIPK8 are currently known to regulate salinity tolerance by helping to maintain Na^+^ homeostasis^14^. Several CIPKs are also known to regulate K^+^ homeostasis in response to abiotic stresses, including salinity and low potassium conditions^15^.

To counteract salinity stress, plants employ mechanisms that regulate intracellular Na^+^ concentration through active efflux of Na^+^ across the plasma membrane and vacuolar sequestration of sodium ions. These processes are mediated by ion transporters, particularly Na^+^/H^+^ antiporters (sodium–proton exchangers: NHX proteins), which facilitate the exchange of sodium ions across cellular membranes using proton gradients^16,17^. *A. thaliana* encodes eight NHX isoforms (NHX1 to NHX8). Among these, AtNHX7—also known as salt overly sensitive 1 (SOS1)—is a plasma membrane-localized Na^+^/H^+^ antiporter that plays a central role in Na⁺ efflux^18^. Earlier studies suggested that vacuolar-localized AtNHX1 and AtNHX2 contribute to salinity tolerance by transporting cytosolic Na^+^ into the vacuole^19^. However, subsequent research revealed that AtNHX1 and AtNHX2 primarily function as K^+^/H^+^ exchangers, maintaining intracellular K^+^ and pH homeostasis-key processes that support cell expansion, tissue elongation, and stomatal regulation via K^+^ accumulation^20,21^. Additionally, other components such as CBL10 and SOS2 have been implicated in vacuolar Na^+^ sequestration, possibly through regulation of yet unidentified Na^+^ transporters^22^.

Multiple molecular components and signaling pathways contribute to plant salinity tolerance^10^. Among them, the Salt Overly Sensitive (SOS) pathway plays a primary role in providing salinity tolerance in plants by regulating the exclusion of excess sodium from the cell^23^. When plants encounter salinity, the extracellular salt sensor monocation-induced [Ca^2+^]_i_ increase 1 (MOCA1) plays a role in detecting Na^+^ and a few other monovalent cations^24^. MOCA1 is responsible for the biosynthesis of glycosyl inositol phosphorylceramide (GIPC) sphingolipids in the plasma membrane. Binding of Na^+^ to GIPC is proposed to cause depolarization of the cell-surface potential, which in turn activates calcium influx channels and raises intracellular Ca^2+^ levels-triggering the SOS signaling cascade^25^.

The SOS pathway was first discovered in *A. thaliana* and primarily consists of three components: SOS1 (a Na^+^/H^+^ antiporter); SOS2, a CIPK family protein; and SOS3, a CBL protein^9,18,26,27^. In response to elevated cellular Ca^2+^ levels triggered by elevated salinity, SOS3 binds Ca^2+^ and subsequently, calcium-bound SOS3 interacts with and activates SOS2. Activated SOS2 then phosphorylates SOS1, facilitating transport of Na^+^ from inside the cell to outside the cell^28,29^. Under both low and high salt stress conditions, SOS3, SOS2, and SOS1 play a role in salinity tolerance. Under high-salt stress, additional components such as CBL8 (which partners with SOS2 and SOS1) and the CBL10–CIPK8 module also become involved, indicating a layered regulatory mechanism that enhances plant resilience under high salt conditions^14^.

SOS2, a protein kinase that belongs to the CIPK family, plays a critical role in the salt stress signaling pathway^9^. CIPKs contain a conserved N-terminal protein kinase domain responsible for their catalytic activity. Within this domain, the activation loop-typically 20–30 amino acids in length-extends from DFG motif (Asp-Phe-Gly) to the APE motif (Ala-Pro-Glu)^30^. The activation loop is essential for autophosphorylation, which converts a protein kinase from its inactive state to an active state^30,31^. Another defining feature of CIPKs is the NAF motif (also known as the FISL motif; Pfam # PF03822), which serves as a distinctive self-inhibitory motif^32^. The NAF motif inhibits the kinase activity of CIPK by directly interacting with its catalytic domain^33^. Upon stress-induced elevation of intracellular Ca^2^⁺, calcium-bound CBL proteins bind to the NAF motif, disrupting its inhibitory interaction and activating the CIPK. This CBL–NAF interaction is essential not only for relieving autoinhibition but also for the formation of functional CBL–CIPK complexes, such as the SOS3–SOS2 interaction crucial for salinity tolerance^34,35^.

Maintaining potassium (K^+^) homeostasis is a significant factor in plants’ salinity tolerance and a determining element in their survival under saline conditions. A variety of K^+^ channels, such as AKT1, and potassium transporters, such as HAK5, are essential for this K^+^ homeostasis^36,37^. Various factors regulate K^+^ uptake in plants, including membrane potential and the SOS signaling pathway components, which control both high- and low-affinity uptake systems. Additionally, calcium signaling, through the action of CBL and CIPK proteins, plays a key role in K^+^ uptake^38,39^.

Alfalfa (*Medicago sativa*) is an economically important perennial legume forage crop. It is commonly referred to as the “Queen of Forages” due to its ability to produce nutritious and palatable forage under diverse climatic and soil conditions. Importantly, alfalfa hay can contain more than 20% protein^40,41^. In the United States, more than 15 million acres are dedicated to alfalfa cultivation^42^. Salinity severely impairs growth of alfalfa and productivity. Salinity’s detrimental effects include inhibiting seed germination, negatively impacting root system architecture, causing the loss of vital nutrients like potassium, elevating sodium to toxic levels, and suppressing shoot development, including both stems and leaves^17,43^. Although alfalfa is generally considered a moderately salt-tolerant species, substantial variation in salinity tolerance exists among different alfalfa cultivars. A previous study has evaluated the salinity responses of multiple *M. sativa* genotypes by assessing growth traits, ion accumulation, and the expression of key salinity-related genes such as *MsSOS1*, *MsSOS2*, and *MsSOS3*, which are presumed to play roles in ion homeostasis and salt tolerance^4^. Transcriptomic analyses across various alfalfa genotypes have revealed differential regulations of numerous signaling pathways and molecular components associated with salinity stress^44–46^. In particular, genotype-specific expressions of *SOS* genes of *M. sativa* have been reported^4^ and a full-length transcript sequencing study in alfalfa roots further confirmed the activation of the SOS core pathway alongside other stress-responsive pathways^47^. Despite these advances, none of the core SOS signaling components in *M. sativa*—such as *MsSOS1*, *MsSOS2*, or *MsSOS3*—have been functionally validated to date, highlighting a key gap in understanding the molecular basis of salinity tolerance in this species.

This study focused on characterizing *MsSOS2*, a key component of the SOS signaling pathway in *M. sativa*. Sequence analysis confirmed that MsSOS2 contains all conserved domains necessary for kinase function and shares strong homology with SOS2 proteins from Arabidopsis and rice. Protein interaction assays showed that MsSOS2 interacts with MsSOS3, supporting its role in the SOS regulatory complex. Functional complementation of the Arabidopsis *sos2* mutant (hereafter *sos2*) with *MsSOS2* restored salinity tolerance during seed germination, seedling growth, and at the late vegetative stage. Additionally, *MsSOS2*-expressing lines maintained improved Na⁺ and K⁺ balance under salt stress, indicating that *MsSOS2* contributes to ionic homeostasis and enhances plant resilience to salinity.

## Results

### Computational analysis of MsSOS2 protein sequence

We identified and cloned the full-length *MsSOS2* cDNA from a salt-tolerant *M. sativa* genotype SISA 14. The cloned cDNA was 1341 bp in length and encoded a protein of 446 amino acids, consistent with the sequence available in the *Medicago* database (https://medicago.legumeinfo.org/), with no observed variation. The predicted molecular weight of the MsSOS2 protein is 50.8 kDa, with an isoelectric point (pI) of 9.2.

**Fig. 1:**
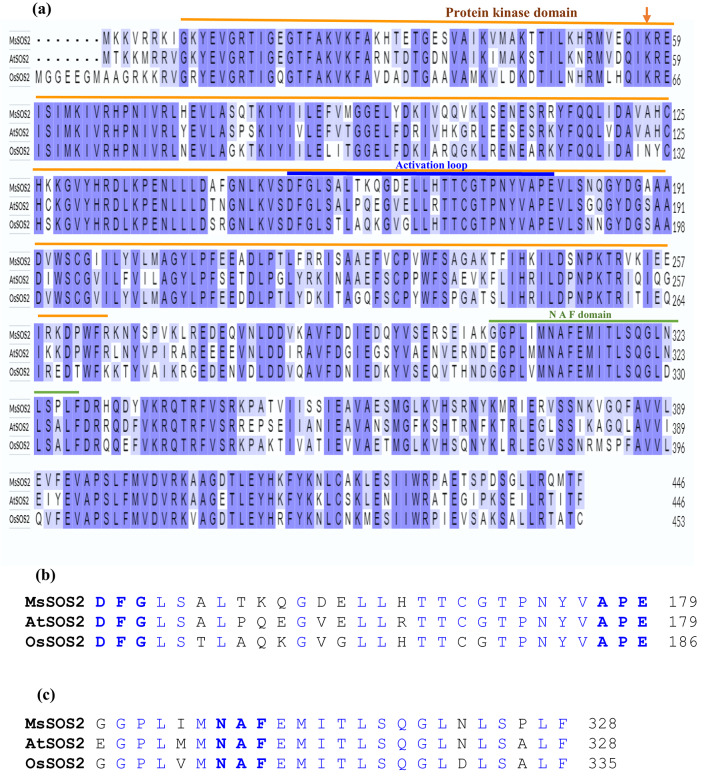
Alignment of MsSOS2, AtSOS2, and OsSOS2 protein sequences. (**a**) The protein kinase domain of MsSOS2 is conserved, as indicated by an orange line. An activation loop is marked with a blue line, and an NAF domain is indicated by a green line. An orange arrow within the protein kinase domain highlights that K57 (Lysine-57), the phosphatidic acid (PA) binding site, is conserved in MsSOS2, similar to AtSOS2 and OsSOS2. (**b**) Activation loop sequences of the protein kinase domain of MsSOS2, AtSOS2, and OsSOS2. Conserved amino acid sequences are shown in blue. (**c**) An NAF domain sequences of MsSOS2, AtSOS2, and OsSOS2, with conserved amino acid sequences also shown in blue.

The SOS2 protein belongs to the CIPK family. All CIPKs are expected to have a protein kinase domain, an ‘activation loop’ within the protein kinase domain, and a ‘NAF’ domain. Protein sequence alignment of MsSOS2 with *Arabidopsis thaliana* SOS2 (AtSOS2) and *Oryza sativa* SOS2 (OsSOS2) revealed that MsSOS2 has a conserved protein kinase domain (9–265) (Fig. 1a). Furthermore, the phosphatidic acid (PA) binding site, Lysine57 (K57) of MsSOS2, critical for membrane localization under salt stress, is conserved across all three species (orange arrow, Fig. 1a). This membrane localization promotes the interaction of SOS2 with SOS1, a step necessary for Na⁺ extrusion under salt stress. In addition, the activation loop, extending from the DFG motif to the APE motif (152–179) is also conserved, supporting its role in kinase activation (Fig. 1a,b). Furthermore, the C-terminal NAF motif (305–328), a hallmark of CIPKs required for interaction with CBL proteins, is present and well conserved in MsSOS2 (Fig. 1a,c). These findings confirm that the MsSOS2 protein retains all defining structural features of a functional SOS2 kinase.

**Fig. 2:**
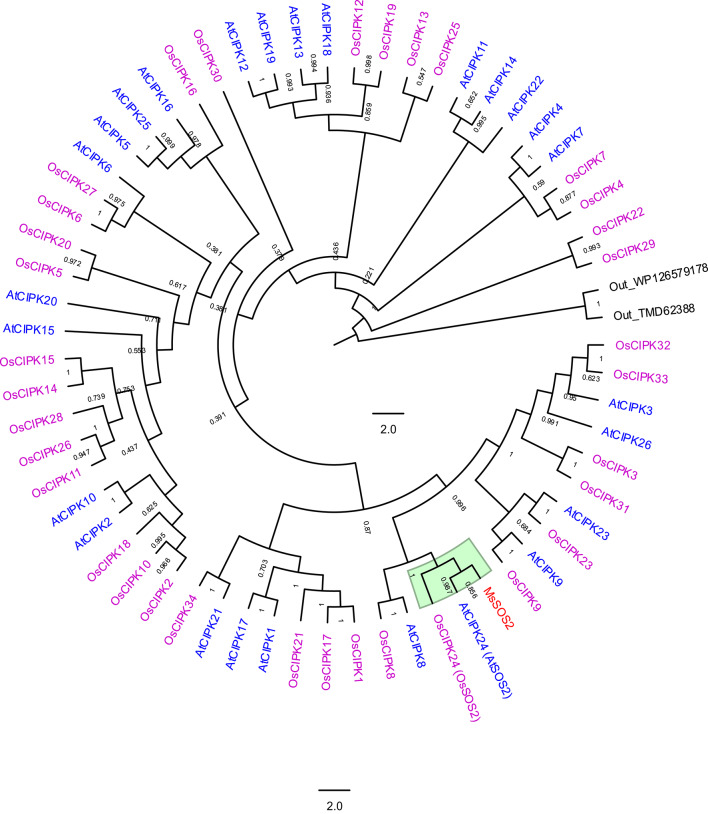
Phylogenetic analysis of MsSOS2 along with CIPKs of *A. thaliana* and *O. sativa*. MsSOS2 is shown in red, while CIPKs of *A. thaliana* and *O. sativa* are represented in blue and magenta fonts, respectively. The SOS2 subclade is highlighted in green. Clade posterior probabilities are shown at nodes. WP126579178 represents a Serine/Threonine protein kinase from *Tengunoibacter tsumagoiensis*, and TMD62388 represents a Ser/Thr protein kinase from Chloroflexota bacterium.

To investigate whether the gene we cloned, *MsSOS2*, represents the structural SOS2 ortholog in *M. sativa*, we conducted a phylogenetic analysis including all 26 Arabidopsis CIPK proteins and 34 rice CIPK proteins. While both species have a single CIPK (AtSOS2 and OsSOS2) involved in SOS signaling pathway, MsSOS2 was found to cluster with these orthologs (Fig. 2). Specifically, MsSOS2 grouped with AtSOS2 and OsSOS2 in a well-supported subclade with high bootstrap values, indicating strong evolutionary conservation (Fig. 2). Notably, MsSOS2 showed closer relationship to AtSOS2 than to OsSOS2 (Fig. 2), suggesting greater sequence similarity with the dicot counterpart. These findings support the identity of MsSOS2, as the structural SOS2 homolog in *M. sativa*.

### MsSOS2 directly interacts with MsSOS3

**Fig. 3:**
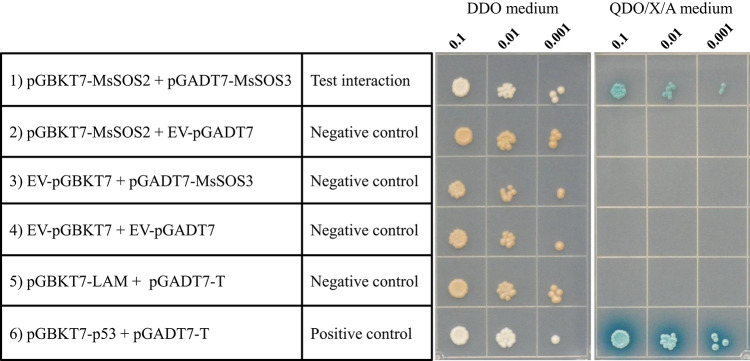
MsSOS2 interacts with MsSOS3 in yeast. Pairwise protein–protein interactions were performed between the specified proteins employing the yeast two-hybrid assay. Transformed yeast clones containing both pGBKT7 and pGADT7 constructs were examined in three tenfold serial dilutions (OD 600 = 0.1, 0.01, and 0.001) on double dropout (DDO) media, SD/-Leu/-Trp. The interaction status of MsSOS2 with MsSOS3, evaluated by the growth assay of yeast cells on interaction-selective Quadruple Dropout media containing X-α-Gal and Aureobasidin (XDO/X/A), SD/-Leu/-Trp/-His/X/A. Empty vectors (EV) were transformed to check the auto-activation status. The Lam and T-antigen protein pair was used as a negative control, while the P53 and T-antigen protein pair was used as positive control.

To determine if the *MsSOS2* gene is a functional *SOS2* ortholog, we hypothesized that it should interact with MsSOS3, analogous to the well-characterized interaction between AtSOS2 and AtSOS3. A yeast two-hybrid assay confirmed that MsSOS2 directly interacts with MsSOS3 (Fig. 3). This finding indicates that the cloned MsSOS2 and MsSOS3 are likely orthologs of AtSOS2 and AtSOS3, respectively and that they retain conserved interaction capabilities essential for SOS signaling.

### *MsSOS2* is induced in response to salinity in *M. sativa* plants

**Fig. 4:**
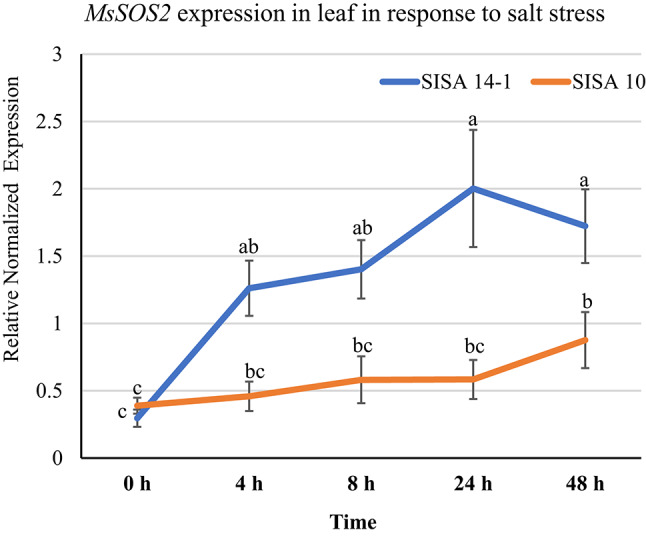
Expression analysis of the *MsSOS2* gene in a salt-tolerant genotype (‘SISA 14-1’) and a salt-sensitive genotype (‘SISA10’) in response to salinity, using RT-qPCR. In this experiment, 10 leaves were used per biological replicate, and five independent biological replicates were used (n = 5). The *y*-axis shows relative normalized expression, while the *x-*axis indicates the expression levels of *MsSOS2* from 0 to 48 h in the leaves of the ‘SISA 14-1’ (blue) and the ‘SISA 10’ (orange) alfalfa plants, respectively. Error bars represent the standard error (± SE). For statistical analysis, a two-way ANOVA and a Tukey’s HSD test were used to show statistical differences in *MsSOS2* expression at indicated time points, both within and between the two lines. Different letters above the error bars indicate a significant difference (*P* < 0.05), while the same letters indicate no significant difference.

To investigate whether *MsSOS2* is regulated at the transcriptional level in response to salinity, we performed time kinetics of *MsSOS2* expression from 0 to 48 h. We used both a salt-tolerant genotype (SISA 14-1) and a salt-sensitive genotype (SISA 10), treating them with either a control solution or a saline solution. *MsSOS2* expression was analyzed by RT-qPCR. Our results revealed that the expression of *MsSOS2* was induced at 4 h in the tolerant genotype, whereas significant induction in the sensitive genotype was observed at 48 h (Fig. 4). Additionally, the expression level of *MsSOS2* in the tolerant genotype was significantly higher from 24 to 48 h compared to the sensitive genotype (Fig. 4). The quicker and higher expression of *MsSOS2* suggests a potential role for it in mediating salinity tolerance in the *M. sativa* tolerant genotype ‘SISA 14-1’.

### Verification of the transgenic lines

A homozygous *A. thaliana* T-DNA insertional *sos2* mutant line (*sos2*), Salk_056101, previously used by other researchers, was used to generate two independent homozygous constitutive *MsSOS2* expressing lines: *MsSOS2* 4-7 and *MsSOS2* 9-7 (see “Materials and methods”). Genotyping PCR confirmed the background of Col-0 (wild-type), the *sos2* mutant, and the two MsSOS2-expression lines. Using the Salk_056101 T-DNA specific primer pairs (LB + RP) amplification was observed in the *sos2* mutant, and both transgenic lines, but not in Col-0, confirming the presence of the T-DNA insertion (Fig. S1a). In contrast, amplification of gene specific primers (LP + RP) occurred only in Col-0 (wild-type), and was absent in *sos2*, and two transgenic lines (*MsSOS2* 4-7, and *MsSOS2* 9-7), indicating that the *sos2* mutant used in this study is homozygous and that both transgenic lines were generated in this homozygous *sos2* mutant background (Fig. S1b). In addition, the *MsSOS2* gene specific primers amplified a product only in the transgenic lines, confirming the presence of the *MsSOS2* transgene (Fig. S1c). These results collectively verify that the two *MsSOS2*-expressing lines carry the transgene and were established in a homozygous *sos2* mutant background.

**Fig. 5:**
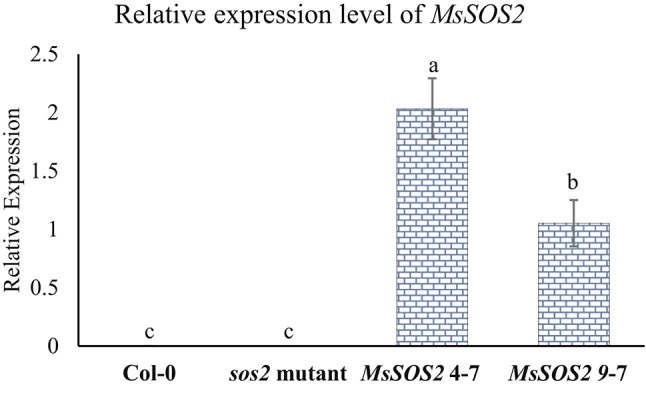
Expression status of the *MsSOS2* gene in Col-0, *sos2* mutant, *MsSOS*2-4-7, and *MsSOS2*-9-7 plants by RT-qPCR. Five leaves were used per replicate, and three biological replicates were used in the assay (n = 3). The *y*-axis shows relative normalized expression of *MsSOS2*, while the *x-*axis indicates the expression levels of *MsSOS2* in the leaves of the indicated genotypes. Error bars represent the standard error (± SE). For statistical analysis, a two-way ANOVA and a Tukey’s HSD test were used to show statistical differences in *MsSOS2* expression between indicated genotypes, and different letters above the error bars indicate a significant difference (*P* < 0.05), while the same letters indicate no significant difference.

The RT-qPCR data confirmed the expression of *MsSOS2* in two transgenic lines, *MsSOS2* 4-7 and *MsSOS2* 9-7 (Fig. 5). As expected, no expression was detected in Col-0 or the *sos2* mutant. Among the transgenic lines, the *MsSOS2* expression was 1.9-fold higher in *MsSOS2* 4-7 compared to *MsSOS2* 9-7 (Fig. 5).

### *MsSOS2* expression in the *sos2* background promotes seed germination under salinity

**Fig. 6:**
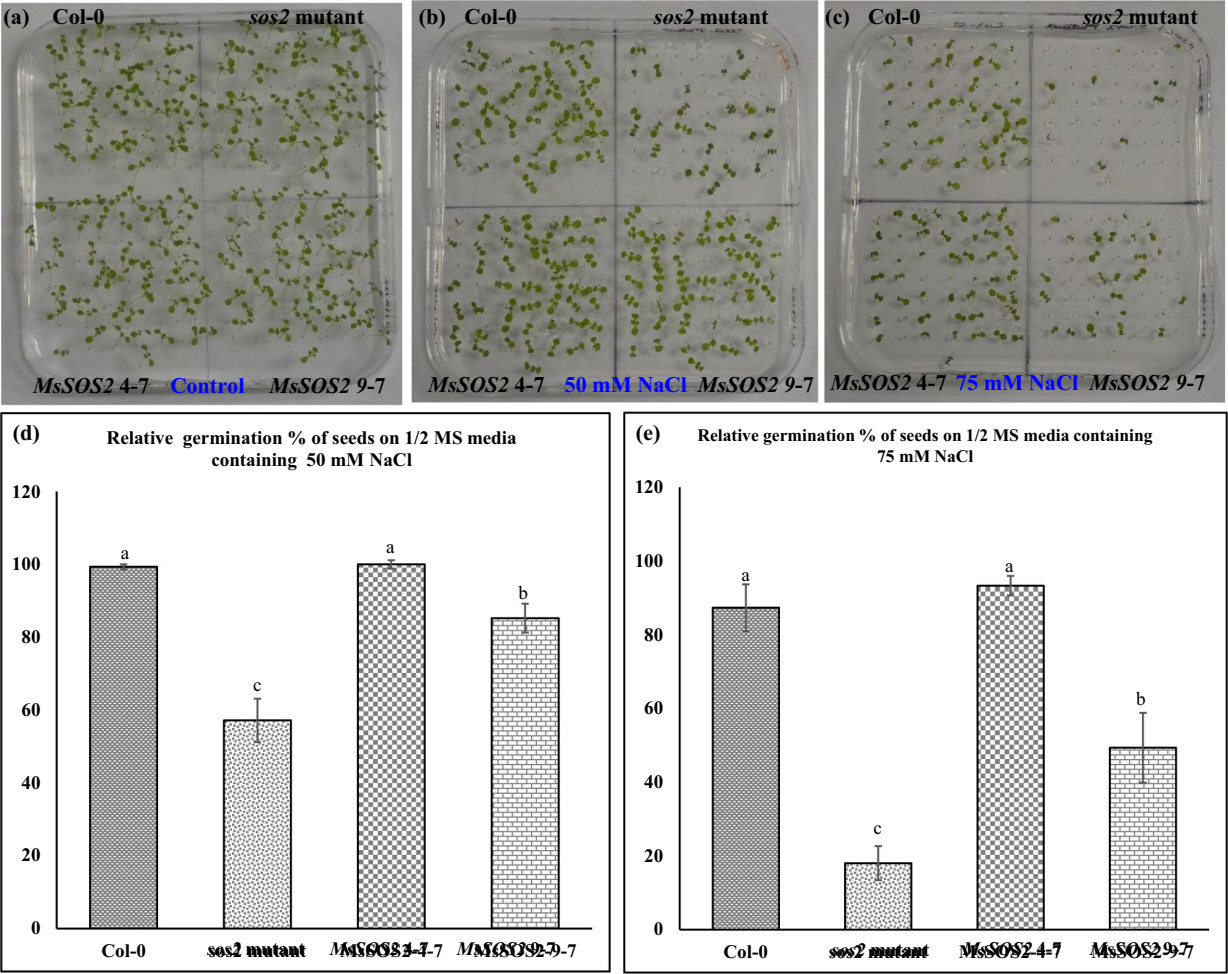
*MsSOS2* positively regulates seed germination in response to salinity. Fifty seeds were used per replicate, and three biological replicates were used in the assay (n = 3). (**a**) Seed germination in a control environment on a ½ MS-Agar plate. (**b**) Seed germination on ½ MS-Agar plates containing 50 mM NaCl. (**c**) Seed germination on ½ MS-Agar plates containing 75 mM NaCl. (**d**) Relative percentage of seed germination for the indicated genotypes under 50 mM NaCl and (**e**) Relative percentage of seed germination for the indicated genotypes under 75 mM NaCl. Error bars represent the standard error (± SE). For statistical analysis (**d**,**e**), a two-way ANOVA and a Tukey’s HSD test were used to show statistical differences in relative germination % of seeds between indicated genotypes (**d**,**e**), different letters above the error bars indicate a significant difference (*P* < 0.05), while the same letters indicate no significant difference.

To evaluate whether the expression of *MsSOS2* could enhance salinity tolerance in the *sos2* mutant, we first assessed seed germination under salt stress. On the ½ MS-Agar plates containing 50 mM NaCl, relative germination rates were 99.3% for Col-0, 57.2% for *sos2* mutant, 100.0% for *MsSOS2* 4-7, and 85.2% for *MsSOS2* 9-7 (Fig. 6a,b,d). At 75 mM NaCl germination rates dropped to 87.3% for Col-0, 18.0% for *sos2* mutant, 93.3% for *MsSOS2* 4-7, and 49.4% for *MsSOS2* 9-7 (Fig. 6a,c,e). These data indicated that the *sos2* mutant exhibited the lowest germination under salinity, while both transgenic lines outperformed the mutant. *MsSOS2* 4-7 showed statistically similar seed germination to Col-0, whereas the *MsSOS2* 9-7 line exhibited lower germination than Col-0 but higher than *sos2* (Fig. 6). Moreover, *MsSOS2* 4-7 consistently outperformed *MsSOS2* 9-7. Our findings demonstrate that heterologous expression of *MsSOS2* enhances seed germination under salinity in the *sos2* background.

### *MsSOS2* expression in the *sos2* background enhances seedling growth under salinity

**Fig. 7:**
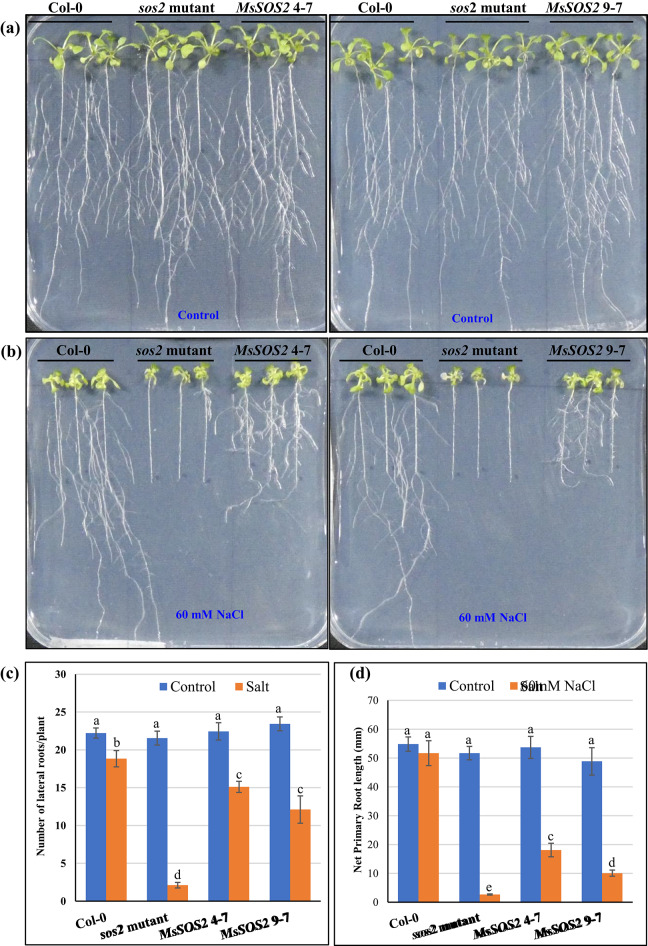
Effect of salinity on seedling growth and lateral root development. Three seedlings of the indicated genotypes were grown per replicate on control or 60 mM NaCl media to quantify lateral root numbers and net primary root length, with five biological replicates used in the assay (n = 5). (**a**) Seedling growth of the indicated genotypes (Col-0, *sos2* mutant, and two homozygous *MsSOS2* transgenic lines: 4–7 and 9–7) is shown on control ½ MS-Agar media. (**b**) Seedling growth on ½ MS-Agar media containing 60 mM NaCl. (**c**) The number of lateral roots per plant for each genotype, grown on control media (blue bars) and media containing 60 mM NaCl (orange bars), is also presented. Error bars represent the standard error (± SE). For statistical analysis, a two-way ANOVA followed by a Tukey’s HSD test was used to determine significant differences in lateral root numbers (**c**) and net primary root length (**d**) between and among the indicated genotypes. In panels (**c**) and (**d**), different letters above the error bars indicate a significant difference (*P* < 0.05), whereas the same letters indicate no significant difference.

We next examined whether the expression of *MsSOS2* could increase seedling growth in response to salinity in the *sos2* mutant background. Under control conditions, seedlings of all four genotypes showed comparable seedling growth, including similar number of lateral roots (Fig. 7a,c). However, in response to salinity (60 mM NaCl), *sos2* exhibited the greatest growth inhibition, while Col-0 displayed the least (Fig. 7b–d). Both transgenic lines outperformed the *sos2* mutant under salinity, showing improved primary root length and lateral root development (Fig. 7b–d). Among the transgenic lines, *MsSOS2* 4-7 seedlings performed better than *MsSOS2* 9-7, including longer primary roots and more lateral roots, although the difference in lateral root numbers was not statistically significant (Fig. 7b–d). As shown in Fig. 7c,d, the *MsSOS2* 4-7 and *MsSOS2* 9-7 transgenic lines displayed significantly reduced primary root growth and lesser number of lateral roots compared to Col-0 plants under salinity stress. This observation suggests that the expression of *MsSOS2* in the *Arabidopsis sos2* mutant was not sufficient to fully restore root development to wild-type levels under salinity.

### *MsSOS2 *expression in the *sos2* background enhances salinity tolerance during late vegetative stage

**Fig. 8:**
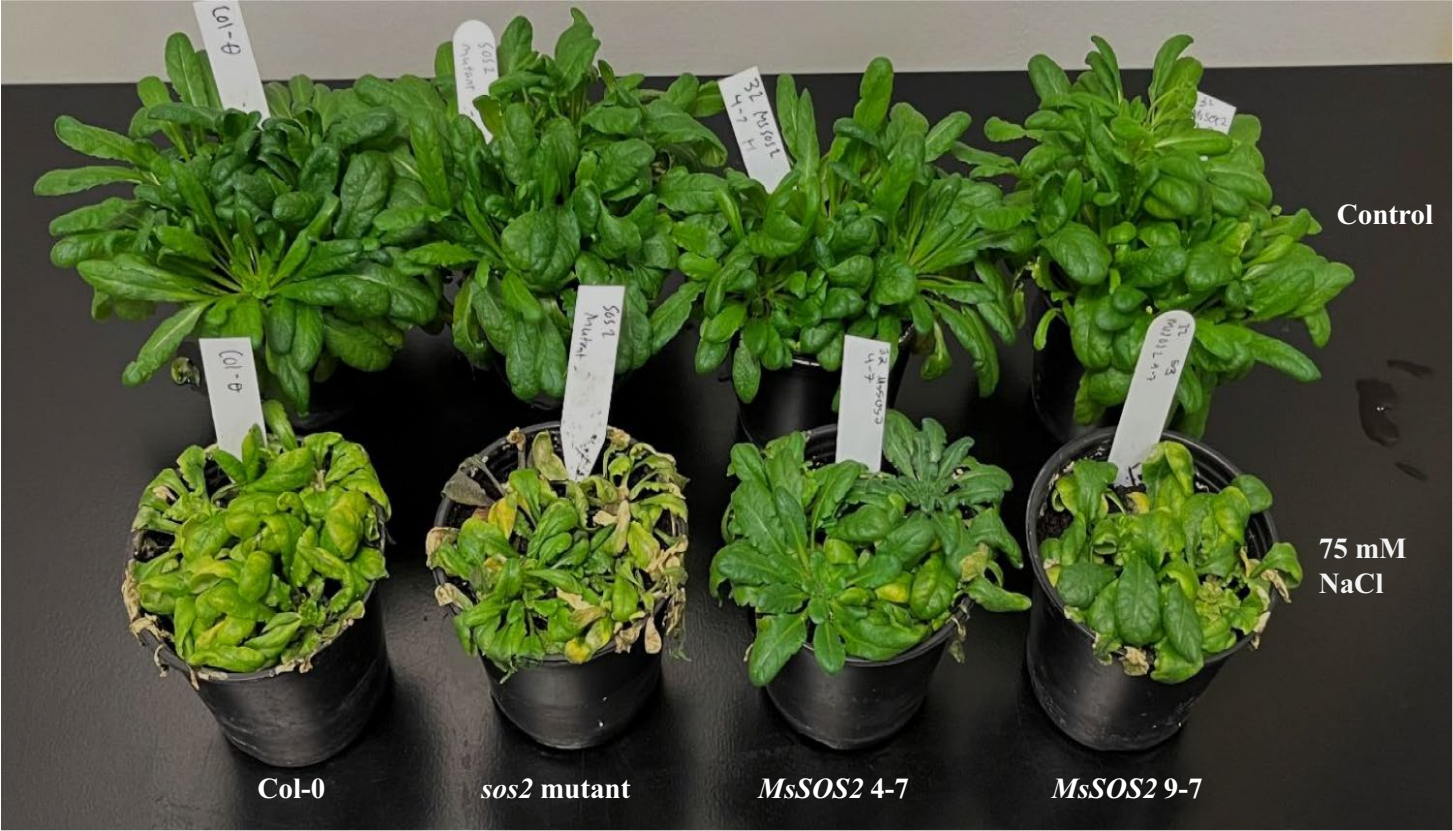
*MsSOS2* expression enhances salt tolerance in the late vegetative stage. Three-week-old Col-0, the *sos2* mutant, and two *MsSOS2* transgenic lines (4-7, 9-7) were treated for 3 weeks. The upper row shows control plants, and the lower row shows plants treated with 75 mM NaCl. The upper row shows that all genotypes grew comparably under control conditions. The lower row clearly indicates that under salt stress, the *sos2* mutant suffered the most severe damage, whereas *MsSOS2* transgenic lines demonstrated improved health and reduced stress symptoms. Notably, *MsSOS2* 4-7 maintained a healthier appearance than *MsSOS2* 9-7.

We also examined whether the expression of *MsSOS2* in the *sos2* mutant background could improve tolerance to salinity at the late vegetative stage. Treatment of three-week-old plants from all genotypes with 75 mM NaCl for an additional three weeks revealed clear differences in salinity tolerance. Under control conditions, plants from all four genotypes appeared more vigorous than their corresponding salt-treated counterparts (Fig. 8). In contrast, salt treatment appeared to inhibit growth in all four genotypes compared to their corresponding controls (Fig. 8). Phenotypically it appears that both transgenic lines showed improved tolerance relative to the *sos2* mutant, with better overall growth and under salt stress, suggesting that *MsSOS2* expression mitigates salt-induced damage (Fig. 8). Notably, *MsSOS2* 4-7 plants appeared healthier and more vigorous than *MsSOS2* 9-7 plants (Fig. 8) suggesting *MsSOS2* 4-7 plants may have better tolerance.

### ***MsSOS2 ***expression in the ***sos2*** background regulates Na^+^ and K^+^ homeostasis

To investigate whether *MsSOS2* expression in the *sos2* mutant could enhance Na⁺ and K⁺ homeostasis under salt stress, 5-week-old Col-0, the *sos2* mutant, and two *MsSOS2*-expressing transgenic lines, *MsSOS2* 4-7 and *MsSOS2* 9-7, were treated with either control solution or a 75 mM NaCl, respectively, for 12 days.

**Fig. 9:**
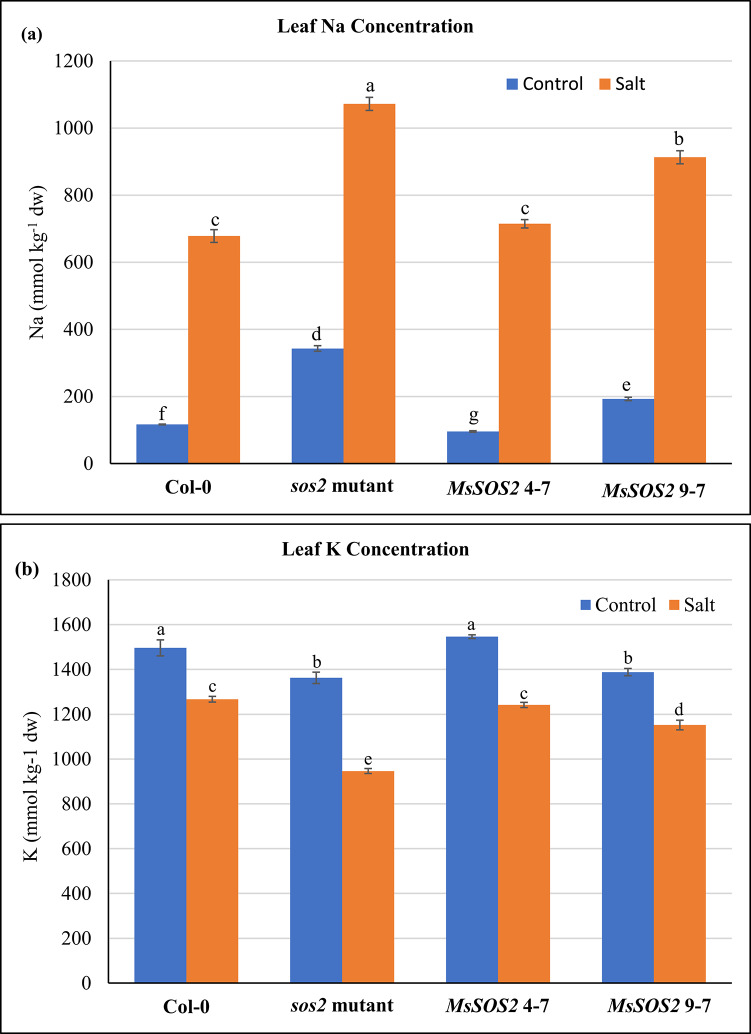
*MsSOS2* positively regulates Na^+^ and K^+^ homeostasis. Control or salt treated leaves were pooled from 12 plants of the indicated genotypes per biological replicate and three biological replicates used to element analyses (n = 3). (**a**) Leaf Na concentration. (**b**) Leaf K concentration. Error bars represent the standard error (± SE). For statistical analysis, a two-way ANOVA followed by a Tukey’s HSD test was used to determine significant differences in leaf Na concentration (**a**) and leaf K concentration (**b**), between and among the indicated genotypes. In panels a and b, different letters above the error bars indicate a significant difference (*P* < 0.05), whereas the same letters indicate no significant difference.

Ion data revealed that under control treatment, the *sos2* mutant accumulated significantly more leaf Na than Col-0 and both transgenic lines (Fig. 9a). *MsSOS2* 4-7 had the lowest Na content, followed by Col-0, while *MsSOS2* 9-7 showed intermediate levels—higher than Col-0 but lower than the *sos2* mutant (Fig. 9a). Under salinity, Na accumulation increased across all genotypes, with the *sos2* mutant exhibiting the highest levels. *MsSOS2 4-7* maintained Na levels comparable to Col-0, while *MsSOS2* 9-7 again showed intermediate accumulation. These results indicate that *MsSOS2* expression reduces Na accumulation, contributing to improved salt tolerance.

In contrast to Na, K concentration declined in all genotypes in response to salt stress (Fig. 9b). Under control and salinity conditions, K accumulation was also measured in all four genotypes. Under control conditions, Col-0 and *MsSOS2* 4-7 maintained high K levels, compared to the *sos2* mutant and *MsSOS2* 9-7 (Fig. 9b). Under salinity stress, the *sos2* mutant accumulated the lowest level of K among the four genotypes. Col-0 and *MsSOS2* 4-7 plants accumulated similar levels of K, which were significantly higher than those observed in the *MsSOS2* 9-7 (Fig. 9b). These findings suggest that the expression of *MsSOS2* in the *sos2* mutant enhanced K accumulation under salinity stress, contributing to salinity tolerance.

## Discussion

This research marks the first report on the functional characterization of *MsSOS2*, a signaling component of the *M. sativa* SOS pathway. Our work details the cloning and characterization of the *MsSOS2* gene and demonstrated its functional relevance in conferring salinity tolerance, thereby establishing its role as a bona fide *SOS2* homolog in *M. sativa*.

The decision to focus on *MsSOS2* was based on the central role of SOS2 in the SOS pathway and its broader interaction network in other plant species^48,49^. Multiple lines of evidence from our study support that MsSOS2 is a CIPK family member and a conserved component of the SOS signaling pathway in *M. sativa*: (1) MsSOS2 shows high sequence conservation with the well-characterized CIPKs, AtSOS2 and OsSOS2, as demonstrated by protein sequence alignment (Fig. 1a). (2) MsSOS2 possesses a highly conserved protein kinase domain, including a conserved activation loop, as observed in other CIPKs (Fig. 1a,b). It also retains the conserved PA binding site, consistent with AtSOS2 and OsSOS2 (Fig. 1a). (3) The presence of a conserved NAF motif in MsSOS2, a hallmark of CIPKs, suggests it has potential to interact with CBL proteins (Fig. 1a,c)^14,34^. (4) Phylogenetic analysis places MsSOS2 with SOS2-specific subclade of the CIPK family, clustering closely with AtSOS2 and OsSOS2 (Fig. 2).

We also provide experimental evidence demonstrating that MsSOS2 is a functional protein. Yeast two-hybrid assays confirmed a specific interaction between MsSOS2 and MsSOS3, supporting the functional activity of MsSOS2 and its role in the SOS signaling pathway (Fig. 3). The in-planta function of *MsSOS2* was assessed by comparing the performance of these lines with the *sos2* mutant and wild-type Col-0 across multiple developmental stages, including seed germination, seedling growth, and the late vegetative phase, under salinity stress.

Seed germination assays under salinity stress revealed that both *MsSOS2* transgenic lines (4-7 and 9-7) exhibited significantly higher seed germination rates compared to the *sos2* mutant. Notably, *MsSOS2* 4-7 showed seed germination rates similar to Col-0, while *MsSOS2* 9-7 had lower rates than Col-0 (Fig. 6), a difference likely attributable to their respective *MsSOS2* expression levels (Fig. 5). One of the primary mechanisms is that the expression of *MsSOS2* in the *sos2* mutant improved seed germination by reducing Na^+^ toxicity and enhancing K^+^ homeostasis compared to the *sos2* mutant (Fig. 9). Additionally, in Arabidopsis, known mechanisms for salt tolerance during seed germination include the CBL5-CIPK8/CIPK24-SOS1 pathway. Specifically, CBL5 interacts with CIPK8 and SOS2, playing a positive regulatory role in alleviating salinity-inhibited seed germination through this pathway^50^. Furthermore, the transcription factor ABI5 is a negative regulator of salt-mediated seed germination, and AFP2 (ABI5-binding protein) is essential for salt tolerance during this critical stage^51^. The interaction of SOS2 with AFP2 stabilizes AFP2, which in turn leads to the degradation of ABI5, thereby enhancing seed germination in response to salinity. Given the high sequence similarity and conservation of various SOS2 domains between MsSOS2 and AtSOS2 (Figs. 1 and 2), one could hypothesize that the AtCBL5-MsSOS2-AtSOS1 module might contribute to alleviating salt-inhibited seed germination in *MsSOS2* transgenic lines. Similarly, the MsSOS2-AFP2 module may also contribute to the enhanced seed germination observed in *MsSOS2* transgenic lines under saline conditions. The potential interaction between AtCBL5 and MsSOS2 warrants investigation. Furthermore, it would be beneficial to determine if *Medicago sativa* employs an MsCBL5-MsCIPK8/MsSOS2-MsSOS1 pathway to confer salinity tolerance during alfalfa seed germination.

In their natural environment, plants initially detect salt stress via their roots, with the signal subsequently propagating to their shoots. Both primary and lateral root development and growth are inhibited in response to salinity, a consequence of reduced cell cycle activity within the meristems^7^. The endodermal ABA signaling pathway mediates both the initial quiescence and subsequent recovery of lateral root growth under salt stress^52^. In the *sos2* mutant, salt treatment severely inhibited or eliminated lateral root development. However, expression of *MsSOS2* in the *sos2* mutant background significantly boosted lateral root number compared to the untransformed *sos2* mutant (Fig. 7). This suggests that *MsSOS2* is vital for lateral root development and growth under salt stress. But, in comparison to salt treated Col-0 seedlings, *MsSOS2* expression in the *Arabidopsis sos2* mutant did not completely rescue the density of lateral roots to the level of Col-0 plants under salt stress. Although MsSOS2 and AtSOS2 are orthologs, their protein sequences are 73% identical (Fig. 1). This suggests that the incomplete rescue of lateral root density in the transgenic lines could stem from structural variations in MsSOS2 compared to AtSOS2. Such variations might result in compatibility issues with crucial interacting partners, which are vital for proper lateral root growth and development. Further research is necessary to determine the specific mechanisms by which MsSOS2 regulates lateral root growth under salt stress and why this protein cannot completely rescue lateral root development in the *sos2* mutant. Additionally, compared to salt-treated Col-0 seedlings, we did not observe the complete rescue of primary root growth in either transgenic line in response to salinity (Fig. 7d). This could be due to dissimilarities between the MsSOS2 and AtSOS2 proteins, leading to a compatibility issue with their target partners, or due to different functional aspects of these two proteins. Observations consistent with these findings have been documented in studies where a heterologous *SOS2* gene, such as *PpSOS2*, was expressed in the *Arabidopsis sos2* mutant, resulting in an incomplete rescue of lateral root density and primary root growth under salinity stress^9^. Our observations, consistent with those of other studies, indicate a significant suppression of lateral root emergence in the *sos2* mutant under salinity (Fig. 7). The expression of MsSOS2 in our transgenic lines partially rescued this phenotype, contributing to the development of lateral roots under salt stress. One of the primary functions of SOS2 is to regulate Na^+^ homeostasis, which is critical for lateral root development. Consequently, a pivotal question remains: Does SOS2 exert its influence on lateral root emergence by transcriptionally regulating or phosphorylating key proteins? In Arabidopsis, the receptor-like kinase GSO1 is a key player in salt tolerance, as it helps prevent salinity-induced root growth inhibition. Under salt stress, GSO1 is expressed in the root tip where it activates the SOS pathway by phosphorylating SOS2 at a conserved threonine residue at position 16 (Thr^16^)^53^. This action promotes the efficient removal of Na^+^ via SOS1, and notably, this function is independent of SOS3. It would be interesting to determine whether AtGSO1 could phosphorylate MsSOS2 Thr^16^. If GSO1 could not phosphorylate MsSOS2 at this site, this might be one of the reasons why MsSOS2 could not rescue primary root growth under salinity in *sos2* mutant. It would also be worthwhile to investigate whether the MsGSO1-MsSOS2-MsSOS1 pathway exists in *M. sativa*.

Salinity tolerance was also evident at the late vegetative stage, where both lines exhibited enhanced growth relative to the *sos2* mutant, and *MsSOS2* 4-7 maintained a healthier phenotype than *MsSOS2* 9-7 (Fig. 8). Collectively, these findings demonstrate that *MsSOS2* is a functional ortholog of *AtSOS2*, capable of performing its biological and biochemical role in a heterologous system across multiple developmental stages.

To assess the role of *MsSOS2* in ion homeostasis, we compared Na and K accumulation among the transgenic lines, the *sos2* mutant, and Col-0. Under control conditions, the *sos2* mutant accumulated significantly more Na than Col-0 and both *MsSOS2* lines, likely due to the loss of *AtSOS2* function (Fig. 9a). *MsSOS2* 9-7 showed higher Na levels than *MsSOS*2 4-7, consistent with its lower expression level (Figs. 5, 9a). Under salt stress, both transgenic lines accumulated less Na than the *sos2* mutant, suggesting that *MsSOS2* contributes to Na⁺ regulation, likely in coordination with *AtSOS1*. Importantly, Na levels in *MsSOS2* 4-7 were comparable to Col-0, indicating functional equivalence to *AtSOS2*. These results support the conclusion that *MsSOS2* plays a key role in Na⁺ homeostasis, and that its expression level is a limiting factor under salinity stress.

Potassium homeostasis is essential for plant salinity tolerance, and the SOS pathway has also been implicated in its regulation^54^. Salinity stress led to a significant reduction in K accumulation in all four genotypes: Col-0, *sos2* mutant, *MsSOS2* 4-7, and *MsSOS2* 9-7, compared to their respective controls (Fig. 9b). The most pronounced reduction in K concentration was observed in the *sos2* mutant, likely due to the absence of functional *AtSOS2*. Notably, under saline conditions, *MsSOS2* 4-7 and Col-0 plants maintained similar K accumulation levels, which were significantly higher than those in the *sos2* mutant, suggesting that the *MsSOS2* 4-7 line can restore K^+^ homeostasis similarly to the wild type (Fig. 9b). Furthermore, the *MsSOS2* 9-7 line showed intermediate K accumulation, higher than the *sos2* mutant but lower than Col-0 or *MsSOS2* 4-7, probably due to the lower expression level of *MsSOS2* in this line (Fig. 9b). These findings suggest that *MsSOS2* functions through a mechanism analogous to *AtSOS2*, potentially involving PA binding and CBL10 phosphorylation, both critical for maintaining K⁺ homeostasis under salt stress.

Lipid signaling, particularly via PA, plays an important role in cell signaling in response to a variety of abiotic stresses, including salinity^55^. Among its diverse functions, PA plays a critical role in modulating multiple components of the salinity response pathways to maintain ion homeostasis^10^. During salt stress in Arabidopsis, PA binds to Lys57 of SOS2, anchoring the protein to the plasma membrane. This enables SOS2-SOS1 interaction, activating Na^+^/H^+^ antiporter activity and promoting Na^+^ efflux and homeostasis. PA also contributes to K^+^ homeostasis. Under non-stress conditions, CBL10 (SCaBP8) interacts with and inhibits the activity of AKT1^36^, but under salt stress, PA-activated SOS2 phosphorylates CBL10, releasing its inhibition of AKT1. This activates AKT1, increasing K⁺ uptake and maintaining cellular ionic balance and turgor under saline conditions^36^.

**Fig. 10:**
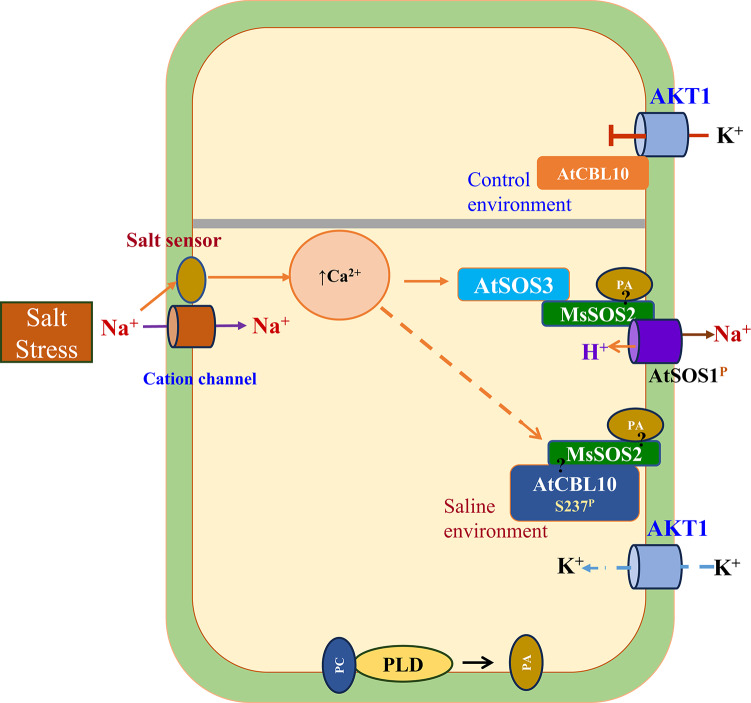
MsSOS2-mediated Na^+^ and K^+^ homeostasis in transgenic Arabidopsis. Under salinity, phospholipase D (PLD) hydrolyzes phosphatidylcholine (PC) to generate phosphatidic acid (PA). Salinity also induces intracellular Ca^2+^, which activates AtSOS3 upon binding. Activated AtSOS3 then facilitates MsSOS2 interaction and activation, concurrently with its PA-mediated membrane anchoring. This combined action subsequently activates AtSOS1, ultimately leading to Na^+^ homeostasis. The upper part of the figure, partitioned with thick grey line illustrates that AtCBL10 inhibits AKT1 under control conditions. In response to salinity, PA-bound MsSOS2 interacts with AtCBL10 and inhibits its interaction with AKT1. This allows AKT1 to absorb K^+^, contributing to K^+^ homeostasis. Dashed lines represent a proposed relationship, but not an experimentally verified one. A question mark indicates where direct interaction between the two indicated elements has not been examined.

Our study provides evidence that MsSOS2, the *M. sativa* ortholog of SOS2, likely functions through a similar mechanism. Conservation of Lys57 in MsSOS2 (Fig. 1a), suggests that it could be similarly activated by PA. Under salt stress, both *MsSOS2* transgenic lines (MsSOS2 4-7 and MsSOS2 9-7) exhibited reduced Na accumulation and increased K accumulation compared to the *sos2* mutant (Fig. 9), supporting a role for MsSOS2 in restoring ion homeostasis. Based on these results, we propose a working model (Fig. 10). Under salt stress, Na⁺ enters cells through non-selective cation channels, triggering a rise in cytosolic Ca^2^⁺. This increase activates AtSOS3 (AtCBL4), which then interacts with and activates MsSOS2. Concurrently, phospholipase D (PLD) converts phosphatidylcholine (PC) to PA, which binds to Lys57 of MsSOS2, a conserved site also found in AtSOS2 and OsSOS2 (Fig. 1a). PA binding facilitates anchoring of MsSOS2 to the plasma membrane and enhances its activity. Activated MsSOS2 phosphorylates AtSOS1, promoting Na⁺ efflux and restoring Na⁺ homeostasis (Fig. 10).

Beyond Na⁺ export, we postulate that PA-bound MsSOS2 contributes to K⁺ homeostasis by phosphorylating AtCBL10 to relieve AKT1 inhibition, permitting K⁺ influx. To test whether MsSOS2 regulates at the transcription level, we quantified *AtSOS1* and *AtAKT1* mRNA in 10-day-old seedlings (Col-0, *sos2*, MsSOS2 4-7, MsSOS2 9-7) after 24 h in 100 mM NaCl; neither gene differed significantly under control or salt (Fig. S2). Together with the known post-translational control of SOS1 by SOS2, these results point to post-translattional regulation as the predominant mode under our conditions. We note this analysis was limited to one dose and time point; broader time-courses, doses, and developmental stages are warranted.

Elements of the MsSOS2–AtCBL10–AKT1 branch remain hypothetical in our system: direct PA binding to MsSOS2 and MsSOS2–AtCBL10 interaction were not tested, and speculative steps are marked with dashed arrows/question marks in Fig. 10. In *M. sativa*, the existence and contribution of a native MsSOS2–MsCBL10–MsAKT1 module to K homeostasis are unknown; additional inward K channels/transporters may also participate. Future work should include biochemical interaction/phosphorylation assays, AKT1 activity readouts, and systems-level transcriptomic/proteomic analyses to define MsSOS2’s broader regulatory network.

## Materials and methods

### Identification of protein kinase domain, activation loop and NAF domain of MsSOS2

The MEGA11 software was used for alignment MsSOS2, AtSOS2 and OsSOS2 protein sequences to identify protein kinase domain (IPR000719), the activation loop, the NAF/FISL domain (IPR004041, NAF motif (IPR018451), and phosphatidic acid (PA) binding site^14^.

### Identification and cloning of the *MsSOS2* and* MsSOS3* genes

Full-length cDNA sequences of *MsSOS2* and *MsSOS3* were obtained by performing protein sequence BLAST analysis using Arabidopsis *AtSOS2* and *AtSOS3* sequences, respectively (https://medicago.legumeinfo.org/). The full-length ORFs of *MsSOS2* and *MsSOS3* were amplified from salt-tolerant alfalfa genotype SISA14 cDNA and cloned using gene-specific primers (Table S1).

Total RNA was isolated from *M. sativa* (‘SISA 14-1’) using Trizol^4^. Subsequently, the isolated total RNA was treated with DNase I (NEB, Ipswich, MA, USA). Then, first-strand cDNA was synthesized using the Invitrogen SuperScript IV Kit (Invitrogen, Waltham, CA, USA). The full-length *MsSOS2* and *MsSOS3* genes were PCR amplified from *M. sativa* cDNA using Q5 High-Fidelity DNA polymerase (NEB, Ipswich, MA, USA) and gene-specific primers (Table S1).

PCR products of *MsSOS2* and *MsSOS3* were column purified, and subsequently, an A-tailing reaction was performed using standard Taq-polymerase and dATP and incubated at 72 °C for 30 min. Next, the A-tailed PCR products were cloned into the pCR^TM^8/GW/Topo TA cloning vector (Invitrogen, Waltham, CA, USA). Sanger sequencing was performed to verify the sequences. Sequence-verified clones were then used for subcloning into plant or yeast expression vectors.

The Expasy (https://web.expasy.org/) tool was used to compute the theoretical pI and molecular weight of MsSOS2.

### Phylogenetic analysis

The protein sequence of MsSOS2, along with 26 CIPKs from *A. thaliana* and 34 CIPKs from *Oryza sativa*, was used for phylogenetic analysis (Table S2). Two bacterial protein kinases were used as an outgroup as described earlier^14^. MEGA11 was used for sequence alignments, and the ‘Find Best DNA/Protein Models (ML)’ option was selected within the MEGA11 software suite for the analysis. The phylogenetic tree was constructed using the ‘Maximum Likelihood’ method, and its robustness was tested using the Bootstrap method with 1000 bootstrap replications. The JTT model was used for substitution. The FigTree v1.4.4 software was used to generate the final figure.

### Alfalfa propagation, salinity treatment, and tissue sampling

Salt-tolerant (‘SISA 14-1’) and salt-sensitive (‘SISA 10’) alfalfa genotypes were propagated asexually using 10–12 cm cuttings with two nodes. The cut ends were moistened, dipped in TakeRoot rooting hormone (0.1% Indole-3-butyric acid), and placed in 50 mL tubes with tap water inside a growth chamber for 2–3 weeks until roots developed. Twelve rooted cuttings of each genotype were then transplanted into sand tanks in a greenhouse at the US Salinity Laboratory (USDA-ARS) in Riverside and irrigated daily with either control (EC = 1.46 dS m^−1^) or saline solution (EC = 18 dS m^−1^; Table S3).

For gene expression analysis, six healthy plants per genotype were selected from separate tanks. Ten leaves per plant were harvested at 0, 4, 8, 24, and 48 h after initial salinity treatment. Leaf samples were snap-frozen in liquid nitrogen and stored at − 80 °C. Harvested tissues were used for RNA isolation and downstream expression analysis.

### Gene expression analysis

Total RNA was isolated from the indicated tissue using TRIzol reagent (Invitrogen, Waltham, CA, USA) and treated with DNase I (Thermo Fisher Scientific, Waltham, MA, USA) to eliminate DNA contamination. All RT-qPCR primers were designed using Primer-BLAST, a tool that designs target-specific primers (https://www.ncbi.nlm.nih.gov/tools/primer-blast/). RT-qPCR was performed on a BioRad CFXS system (Bio-Rad Laboratories) with the iTaq Universal SYBR Green one-step kit in a 10 μL reaction volume. Each reaction contained 10.0 ng total RNA, 0.125 μL iScript Reverse transcriptase, 5 μL of 2 × SYBR Green mix, and 0.75 μM forward and reverse primers Table S1). The cycling conditions were: 50 °C for 10 min (cDNA synthesis), 95 °C for initial denaturation, followed by 40 cycles of 95 °C for 10 s (denaturation), 57 °C for 30 s (annealing), and 68 °C for 30 s (extension). To examine tissue-specific and salt stress-induced relative expression of *MsSOS2* in *M. sativa* (‘SISA 14-1’), *glyceraldehyde 3-phosphate dehydrogenase* (*G3PD*) and *Actin* (*Act*) were used as reference genes. For Arabidopsis RT-qPCR, *AtUbiquitin5* and *AtActin2* served as reference genes for normalization. Relative expression was determined using the Ct values of target genes normalized to the reference genes.

### Yeast two-hybrid assay (Y2H assay)

The entry clone *MsSOS3* in the pCR8 vector, was sub-cloned into the pGADT7-AD-GW vector, and similarly the entry clone *MsSOS2* in the pCR8 vector was sub-cloned into the pGBKT7-GW vector. Specific pairs of constructs (e.g., pGADT7-GW-MsSOS3 + pGBKT7-GW-MsSOS2) were co-transformed into the *Saccharomyces cerevisiae* Y2HGold strain according to the manufacturer’s protocol (Clontech Laboratories, Mountain View, CA, USA). Co-transformed yeasts were selected on SC-Trp-Leu plates, a double dropout (DDO) medium. Subsequently, to check the interaction status of the two protein pairs (MsSOS3 + MsSOS2), selected co-transformed yeasts were plated on SD-Leu, -Trp, -His, -Ade medium, a quadruple dropout (QDO) medium, supplemented with 5-bromo-4-chloro-3-indolyl-α-D-galactopyranoside (X-α-Gal) or 150 ng/ml of Aureobasidin A (AbA) (QDO/X/A). To check the autoactivation status, each bait and prey construct was co-transformed with the corresponding empty vector used for the Y2H assay. The interaction between T-antigen and P53 proteins was used as a positive control, whereas the interaction between T-antigen and Lam proteins was used as a negative control for the Y2H assay. Colonies that showed a blue color were considered to indicate a positive interaction^14^.

### Description of plant genetic materials and growth conditions

We obtained the *Arabidopsis sos2* mutant, Salk_056101, carrying a T-DNA insertion in the promoter region of the *AtSOS2* gene (AT5G35410), from the Arabidopsis Biological Resource Center (http://www.arabidopsis.org/)^9^. This mutant is in Col-0 background. Col-0 seeds, seedlings, or late vegetative stage plants were used as the wild-type for all comparative studies. The expression level of *AtSOS2* was reported to be significantly knocked down in the *sos2* mutant, Salk_056101, in two previous studies, using semi-quantitative RT-PCR or RT-qPCR^9,56^.

The seeds were surface sterilized with 70% ethanol (5 min) and then with 20% bleach solution containing 0.02% Tween 20 or 1.0 µL/mL of 10% SDS (10 min), with mild shaking during both steps. After sterilization, the seeds were washed 5–7 times with sterilized DI water, also with mild shaking, and then suspended in 0.15% agar before being sown onto half-strength Murashige and Skoog (MS) media (Research Products International, Mount Prospect, IL, USA) supplemented with 1.0% sucrose and 0.55% Agar-Plant (Research Products International, Mount Prospect, IL, USA) using a 1000 µL pipettor. The seeds were then vernalized for 3 days at 4 °C. After vernalization, the MS-agar plates containing vernalized seeds were transferred to a CONVIRON GR96 growth chamber (Winnipeg, MB, Canada) set at 21 °C (8 h day)/18 °C (16 h night), with a light intensity of 150 μmol m⁻^2^ s⁻^1^ and approximately 50% relative humidity, to facilitate seed germination and seedling growth. Similar conditions were also provided in alternative growth chambers or plant growth rooms. Seven to nine days-old seedlings were transplanted to Metro-Mix (Sun Gro Horticulture, Agawam, MA, USA).

### Generation of transgenic* MsSOS2* plants in Arabidopsis *sos2* mutant

The pMDC32, a “Gateway compatible plant expression vector,” was used to express *MsSOS2* cDNA in a homozygous *sos2* mutant (Salk_056101), which is a T-DNA insertion mutant. For this, an error-free *MsSOS2* cDNA clone was subcloned into the pMDC32 vector using Gateway™ LR clonase (Invitrogen, Waltham, MA, USA), following the manufacturer’s instructions. An error-free *pMDC32*-*MsSOS2* construct was transformed into *Agrobacterium tumefaciens* strain GV3101 by electroporation. The *sos2* mutant (Salk_056101) was transformed using the floral dipping method. Transgenic seedlings were selected on ½ MS-agar plates containing 1% sucrose and supplemented with 25 µg/mL Hygromycin. The identity of the *MsSOS2* transgenic seedlings was verified by genotyping PCR using *HPTII*-specific primers and subsequently confirmed again by genotyping PCR using *MsSOS2*-specific primers. Two independent Arabidopsis transgenic lines, each harboring a single transgene copy and exhibiting different expression levels, were used for functional analyses. All experiments were performed using seeds from T3 transgenic lines.

### *MsSOS2* expression in two independent transgenic lines

Leaf tissues were collected from four-week-old *MsSOS2* 4-7, *MsSOS2* 9-7, Col-0, and Arabidopsis *sos2* mutant plants for RNA isolation, which were later used for RT-qPCR analysis. The plants were grown in a growth chamber at 21 °C (8 h day)/18 °C (16 h night) with a light intensity of 150 μmol m⁻^2^ s⁻^1^ and approximately 50% relative humidity. For the RT-qPCR assay, five leaves were used per replicate, and three biological replicates were conducted (n = 3). Col-0 and the Arabidopsis *sos2* mutant served as negative controls. For statistical analysis, a two-way ANOVA and a Tukey’s HSD test were used to determine significant differences in *MsSOS2* expression between the indicated genotypes.

### Salt tolerance assay at germination at stage

Sterilized seeds were sown on ½ MS-Agar plate without NaCl supplementation or supplemented with 50- or 75 mM NaCl. The seeds were then vernalized for 3 days at 4 °C. After vernalization, the MS-agar plates containing vernalized seeds were transferred to a growth chamber. We considered a seed as successfully germinated when it showed both radicle and cotyledons. Germination data were collected on the sixth day post incubation in the growth chamber. Fifty seeds were used per replicate, and three biological replicates were used in the assay (n = 3). For statistical analysis, a two-way ANOVA and a Tukey’s HSD test were used to show statistical differences (*P* < 0.05) in the relative germination % of seeds between different genotypes.

### Salt tolerance assay at seedling stage

For the salinity tolerance assay at the seedling stage, 5-day-old seedlings of Col-0, the *sos2* mutant, and two transgenic lines (*MsSOS2* 4-7 and *MsSOS2* 9-7) were transplanted and grown vertically for seven days on MS-agar plates containing 60 mM NaCl or on control MS-agar plates without NaCl. Lateral root numbers were counted, and net primary root growth was measured on the 7th day post-transplantation on control MS-agar and salt-containing MS-agar plates, with pictures captured the following day. Three seedlings of each genotype were grown per replicate on control or 60 mM NaCl media to quantify lateral root numbers and net primary root length, with five biological replicates used in the assay (n = 5). For statistical analysis, a two-way ANOVA followed by a Tukey’s HSD test (*P* < 0.05) was used to determine significant differences in lateral root numbers and net primary root length between and among the four genotypes.

### Measurement of Na and K in late vegetative stage of plants

To examine the accumulation status of Na and K in late vegetative stage of plants in response to 75 mM NaCl, 5-week-old Col-0, the *sos2* mutant, and two transgenic lines (*MsSOS2* 4-7 and *MsSOS2* 9-7) were treated with 1/8th MS salt solution (control) or NaCl salt solution prepared in 1/8th MS salt solution. The NaCl concentration increased incrementally to 75 mM: 25 mM on the first day, 50 mM on the second day, and 75 mM on the third day. Subsequently, every alternate day, plants were watered with the control solution or the salt solution, respectively. After the 12th day of treatment, control or salt-treated leaves were pooled from 12 plants of each genotype per biological replicate, and three biological replicates were used for element analyses (n = 3). Subsequently, leaf tissues from both control and salt-treated plants were dried at 70 °C for over five days. The dried tissues were then ground. Na and K levels were determined by nitric acid digestion followed by inductively coupled plasma optical emission spectrometry (ICP-OES) using the Optima 8000 Perkin-Elmer Corp. instrument (Waltham, MA, USA). For statistical analysis, a two-way ANOVA followed by a Tukey’s HSD test was used to determine significant differences (*P* < 0.05) in leaf Na concentration and leaf K concentration between and among the four genotypes.

## Supplementary Information

Supplementary Information 1.

Supplementary Information 2.

## Data Availability

The nucleotide sequences of **MsSOS2** (GenBank accession number PV851440) and **MsSOS3** (GenBank accession number PV851441) have been deposited in GenBank. All other data supporting the findings of this study are included in the article and its supplementary materials.

## Abbreviations

aa: Amino acids
CBL: Calcineurin B-like
CIPK: CBL interacting protein kinase
NHX: Na^+^/H^+^ exchanger
PA: Phosphatidic acid
PC: Phosphatidylcholine
PLD: Phospholipase D
QDO: Quadruple dropout
SOS: Salt overly sensitive
