# Supplementary material for: Alfalfa MsSOS2 confers salinity tolerance by promoting lateral root growth and regulating Na+/K+ homeostasis
Source: Sci Rep. 2025 Dec 4;15:43187. doi: 10.1038/s41598-025-21355-1 (PMC12680707; doi:10.1038/s41598-025-21355-1)
Supplement: Supplementary file 1 — Supplementary Information 1. [file 41598_2025_21355_MOESM1_ESM.pdf]

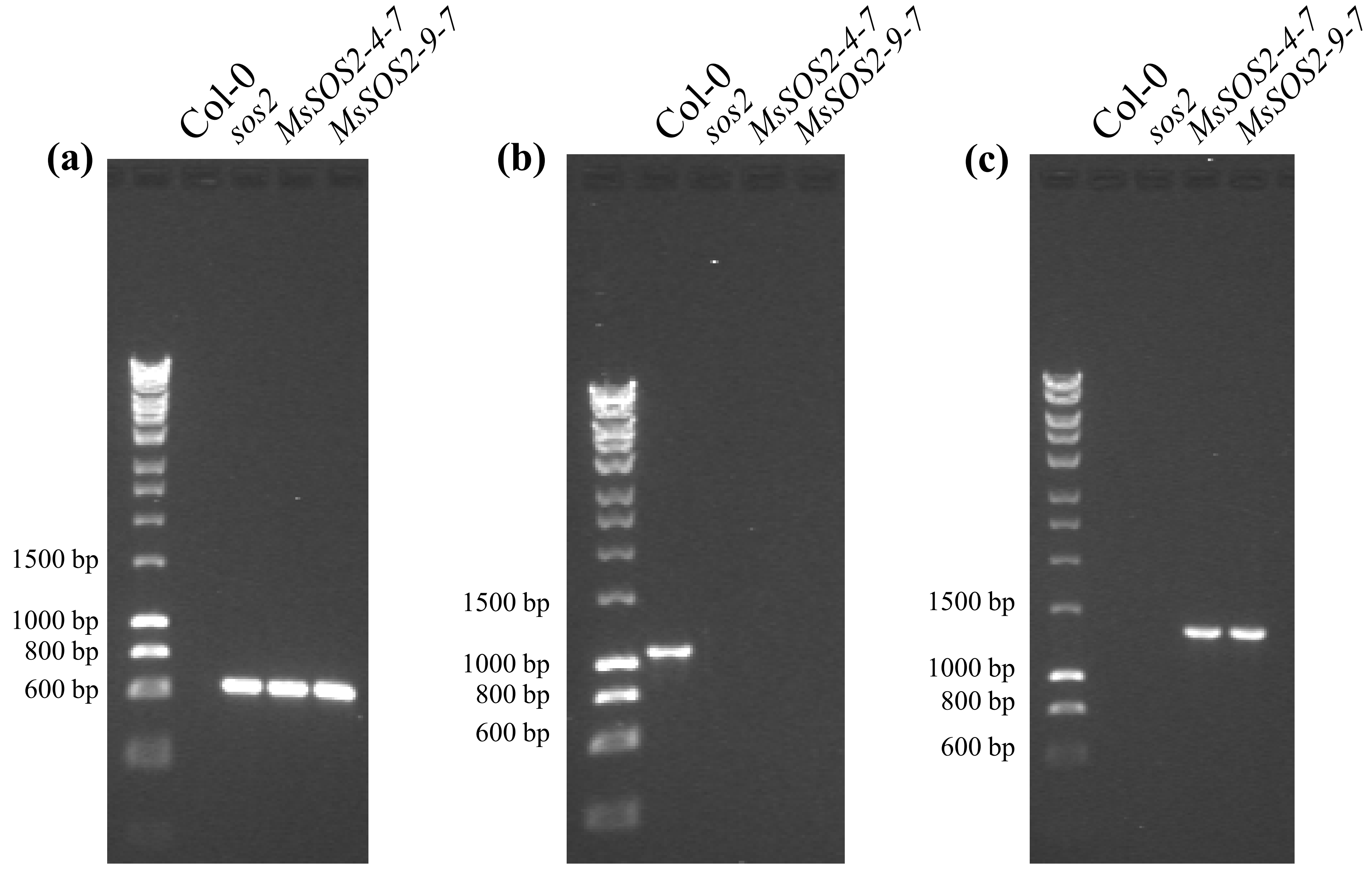

Figure S1. Verification of plant genotypes by genotyping PCR. (a) The *sos2* mutant background was confirmed by amplifying a T-DNA insertion-specific product using LBb1.3 and Salk_056101RP primers. (b) The Col-0 (WT) background was confirmed by genotyping PCR using Salk_056101LP and Salk_056101RP primers. (c) *MsSOS2* transgenic lines were verified by genotyping PCR using *MsSOS2*-specific forward and reverse primers (Table S1).

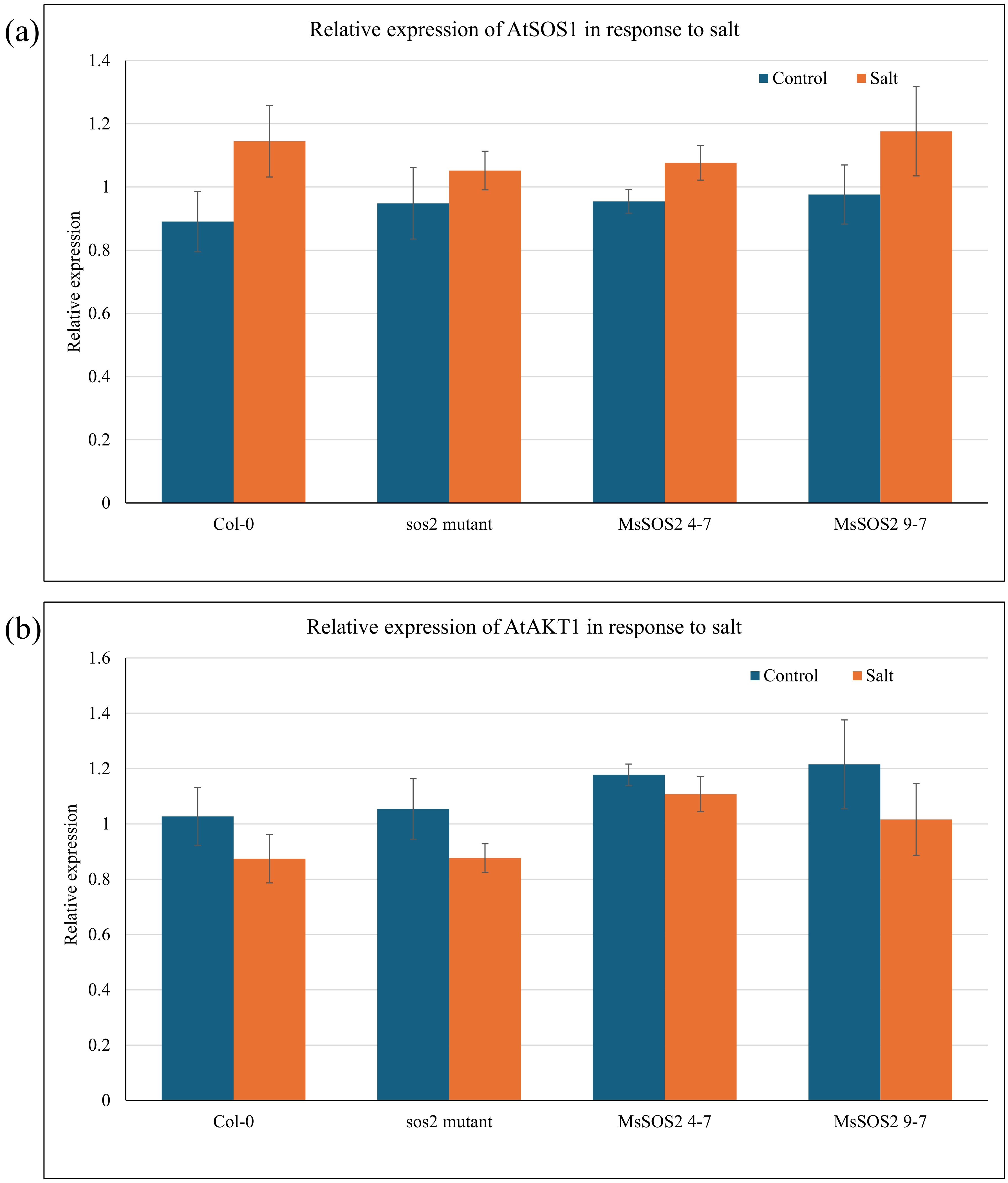

Figure S2. Expression analysis of *AtSOS1* and *AtAKT1 in Col-0, sos2* mutant, and *MsSOS2* transgenic lines, *MsSOS2* 4-7 and *MsSOS2* 9-7. Expression analysis of *AtSOS1* (a) and *AtAKT1* (b) in 10-day-old seedlings of Col-0 (wild type), the *Arabidopsis sos2* mutant, and the *MsSOS2* 4-7 and *MsSOS2* 9-7 transgenic lines. Seedlings were grown for 24 hours on control ½ MS-agar plates or on plates containing 100 mM NaCl. In this experiment, five seedlings were used per biological replicate, with a total of three independent biological replicates (n=3). The y-axis represents relative normalized expression, while the x-axis shows the expression levels of *AtSOS1* (a) and *AtAKT1* (b) under a control condition (blue) and in response to a 24-hour treatment with 100 mM NaCl (orange). Error bars represent the standard error (±SE).
