# Supplementary material for: Alfalfa MsSOS2 confers salinity tolerance by promoting lateral root growth and regulating Na+/K+ homeostasis
Source: Sci Rep. 2025 Dec 4;15:43187. doi: 10.1038/s41598-025-21355-1 (PMC12680707; doi:10.1038/s41598-025-21355-1)
Supplement: Supplementary file 2 — Supplementary Information 2. [file 41598_2025_21355_MOESM2_ESM.docx]

| **Table S1.** List of primers | |
| --- | --- |
| **Primer Name** | **Sequence 5' ── 3'** |
| SALK_056101_LP | GGCCATAAAAGCCTCTTCAAC |
| SALK_056101_RP | TTCCTTCACCTATTGTGCGAC |
| LB 1.3 | ATTTTGCCGATTTCGGAAC |
| MsSOS2-F | ATGAAGAAAGTGAGGAGAAAGATTG |
| MsSOS2-R | TCAGAATGTCATTTGTCGAAGC |
| MsSOS3-F | ATGGGCTGCTTTTGTTCAACTTCAAAGAA |
| MsSOS3-R | TCA ACC CTC TGA GTC TTC AAC TTC TGT TC |
| MsSOS2-RT-qPCR-F | GGTTGAACAGATCAAAAGAGAGA |
| MsSOS2-RT-qPCR-R | CTCCAGAATTATGTAGATCTTGGTC |
| MsActin-RT-qPCR-F | GGAATGGTCAAGGCTGGATTT |
| MsActin-RT-qPCR-R | TGATTGAGCTTCATCACCAACATA |
| MsG3PD-RT-qPCR-F | CATCACAGCCACTCAGAAGAC |
| MsG3PD-RT-qPCR-R | TGGAAGCACTTTGCCTACAG |
| A1SOS1-F | TGCTGAAGGCATTCTCGACAGTG |
| A1SOS1-R | ACTGCGCCCCTCAAACCAGA |
| AtAKT1-F | CAAATACAGGATATGGGAGGCT |
| AtAKT1-R | CCAAACTCGAAAGGCGAAAC |
| AtActin2_RT-qPCR-F | GGTAACATTGTGCTCAGTGGTGG |
| AtActin2_RT-qPCR-R | AACGACCTTAATCTTCATGCTGC |
| AtUBQ5-RT-qPCR-F | CTTACACCAAGCCGAAGAAGA |
| AtUBQ5-RT-qPCR-R | CTCCTTCCTCAAACGCTGAA |

| **Table S2.**  List of the *CIPK* genes used for phylogenetic analysis. | | |
| --- | --- | --- |
| **Species** | **Gene** | **Protein Accession** |
| *Medicago sativa* | *MsSOS2* | MS.gene62401.t1 |
| *Arabidopsis thaliana* | *AtCIPK1* | AT3G17510.1 |
| *Arabidopsis thaliana* | *AtCIPK2* | AT5G07070.1 |
| *Arabidopsis thaliana* | *AtCIPK3* | AT2G26980.4 |
| *Arabidopsis thaliana* | *AtCIPK4* | AT4G14580.1 |
| *Arabidopsis thaliana* | *AtCIPK5* | AT5G10930.1 |
| *Arabidopsis thaliana* | *AtCIPK6* | AT4G30960.1 |
| *Arabidopsis thaliana* | *AtCIPK7* | AT3G23000.1 |
| *Arabidopsis thaliana* | *AtCIPK8* | AT4G24400.1 |
| *Arabidopsis thaliana* | *AtCIPK9* | AT1G01140.3 |
| *Arabidopsis thaliana* | *AtCIPK10* | AT5G58380.1 |
| *Arabidopsis thaliana* | *AtCIPK11* | AT2G30360.1 |
| *Arabidopsis thaliana* | *AtCIPK12* | AT4G18700.1 |
| *Arabidopsis thaliana* | *AtCIPK13* | AT2G34180.1 |
| *Arabidopsis thaliana* | *AtCIPK14* | AT5G01820.1 |
| *Arabidopsis thaliana* | *AtCIPK15* | AT5G01810.1 |
| *Arabidopsis thaliana* | *AtCIPK16* | AT2G25090.1 |
| *Arabidopsis thaliana* | *AtCIPK17* | AT1G48260.1 |
| *Arabidopsis thaliana* | *AtCIPK18* | AT1G29230.1 |
| *Arabidopsis thaliana* | *AtCIPK19* | AT5G45810.1 |
| *Arabidopsis thaliana* | *AtCIPK20* | AT5G45820.1 |
| *Arabidopsis thaliana* | *AtCIPK21* | AT5G57630.1 |
| *Arabidopsis thaliana* | *AtCIPK22* | AT2G38490.1 |
| *Arabidopsis thaliana* | *AtCIPK23* | AT1G30270.1 |
| *Arabidopsis thaliana* | *AtCIPK24/AtSOS2* | AT5G35410.1 |
| *Arabidopsis thaliana* | *AtCIPK25* | AT5G25110.1 |
| *Arabidopsis thaliana* | *AtCIPK26* | AT5G21326.1 |
| *Oryza sativa* | *OsCIPK1* | LOC_Os01g18800.1 |
| *Oryza sativa* | *OsCIPK2* | LOC_Os07g48100.1 |
| *Oryza sativa* | *OsCIPK3* | LOC_Os07g48760.1 |
| *Oryza sativa* | *OsCIPK4* | LOC_Os12g41090.1 |
| *Oryza sativa* | *OsCIPK5* | LOC_Os01g10890.1 |
| *Oryza sativa* | *OsCIPK6* | LOC_Os08g34240.1 |
| *Oryza sativa* | *OsCIPK7* | LOC_Os03g43440.1 |
| *Oryza sativa* | *OsCIPK8* | LOC_Os01g35184.1 |
| *Oryza sativa* | *OsCIPK9* | LOC_Os03g03510.1 |
| *Oryza sativa* | *OsCIPK10* | LOC_Os03g22050.1 |
| *Oryza sativa* | *OsCIPK11* | LOC_Os01g60910.1 |
| *Oryza sativa* | *OsCIPK12* | LOC_Os01g55450.1 |
| *Oryza sativa* | *OsCIPK13* | LOC_Os01g10870.1 |
| *Oryza sativa* | *OsCIPK14* | LOC_Os12g02200.1 |
| *Oryza sativa* | *OsCIPK15* | LOC_Os11g02240.1 |
| *Oryza sativa* | *OsCIPK16* | LOC_Os09g25090.1 |
| *Oryza sativa* | *OsCIPK17* | LOC_Os05g04550.1 |
| *Oryza sativa* | *OsCIPK18* | LOC_Os05g26820.1 |
| *Oryza sativa* | *OsCIPK19* | LOC_Os05g43840.1 |
| *Oryza sativa* | *OsCIPK20* | LOC_Os05g11790.1 |
| *Oryza sativa* | *OsCIPK21* | LOC_Os07g44290.1 |
| *Oryza sativa* | *OsCIPK22* | LOC_Os05g26940.1 |
| *Oryza sativa* | *OsCIPK23* | LOC_Os07g05620.1 |
| *Oryza sativa* | *OsCIPK24* | LOC_Os06g40370.1 |
| *Oryza sativa* | *OsCIPK25* | LOC_Os06g35160.1 |
| *Oryza sativa* | *OsCIPK26* | LOC_Os02g06570.1 |
| *Oryza sativa* | *OsCIPK27* | LOC_Os09g25100.1 |
| *Oryza sativa* | *OsCIPK28* | LOC_Os05g39870.1 |
| *Oryza sativa* | *OsCIPK29* | LOC_Os07g48090.1 |
| *Oryza sativa* | *OsCIPK30* | LOC_Os01g55440.1 |
| *Oryza sativa* | *OsCIPK31* | LOC_Os03g20380.1 |
| *Oryza sativa* | *OsCIPK32* | LOC_Os12g03810.2 |
| *Oryza sativa* | *OsCIPK33* | LOC_Os11g03970.1 |
| *Oryza sativa* | *OsCIPK34* | LOC_Os02g08140.1 |

**Table S3.** Control and treatment irrigation water composition

| **Treatment** | **EC_iw_ dS m^−1^** | **Ion concentration (mmol_c_ L^−1^)** | | | | | | | | |
| --- | --- | --- | --- | --- | --- | --- | --- | --- | --- | --- |
|  |  | **Cl^−^** | **SO_4_^2−^** | **NO_3_^−^** | **PO_4_^3−^** | **HCO_3_^−^** | **Na^+^** | **K^+^** | **Ca^2+^** | **Mg^2+^** |
| **Control** | 1.46 | 1.52 | 2.02 | 5.39 | 1.5 | 3.07 | 1.7 | 6.58 | 3.2 | 2.05 |
| **Saline** | 18 | 147.92 | 31.02 | 5.38 | 1.5 | 3.07 | 121.7 | 6.58 | 34.2 | 26.45 |
